# First-Trimester Pregnancy Loss Due to Condyloma Acuminata: A Twisted Tale of Gravidity

**DOI:** 10.7759/cureus.51847

**Published:** 2024-01-08

**Authors:** Saloni, Jyotsna Potdar, Apoorva Dave, Swati M Dahiphale

**Affiliations:** 1 Obstetrics and Gynecology, Jawaharlal Nehru Medical College, Datta Meghe Institute of Higher Education and Research, Wardha, IND

**Keywords:** sexually transmitted diseases in pregnancy, genital warts, spontaneous abortion, pregnancy, human papilloma virus

## Abstract

The human papillomavirus can induce condyloma acuminata, a benign papillomatous squamous growth with a fibrovascular core that arises in the vaginal canal. These illnesses typically afflict women who are fertile and are frequently encountered during pregnancy, manifesting with a variety of symptoms. The influences of hormones and vaginal secretions cause the lesion to expand quickly during pregnancy. Viral infections are known to be one risk factor for threatening abortions. Infection with human papillomavirus (HPV) during pregnancy has been associated with a risk for spontaneous abortion, preterm delivery, and abnormalities in the placenta. There are many therapeutic approaches available to address the disease; however, it is still unclear which one is the most successful. Additionally, organogenesis is crucial throughout the first trimester, and treatment during this period may elevate the risk of spontaneous abortion. Here, we describe the case of a young woman who experienced vaginal lesions during the first trimester of her pregnancy.

## Introduction

Threatened abortion is characterized by vaginal bleeding occurring before 20 weeks of gestation. It is identified by a closed cervical orifice (os), a positive blood pregnancy test with no expulsion of the products of conception, and no evidence of fetal or embryonic death. The World Health Organization (WHO) defines a threatened abortion as any bleeding during the first half of pregnancy that is not associated with cervical dilatation or bloody vaginal discharge linked to pregnancy. About 25% of pregnant women experience some vaginal bleeding during the first two trimesters; in 50% of these cases, the pregnancy gets terminated. Typically, during a dangerous abortion, there is mild-to-moderate bleeding. Early pregnancy loss is associated with several viral illnesses, such as Epstein-Barr, herpes simplex, mumps, rubella, and parvovirus [1,2].

The benign tumor, known as condyloma acuminata (CA), originates in the vaginal tract and is caused by infection with human papillomavirus (HPV) types 6 and 11. The onset of this illness typically occurs between the ages of 25 and 34, which is the reproductive age, and it could get worse throughout pregnancy. CA mostly causes vaginal secretions, itching, pain, and bleeding; nevertheless, several cases go undiagnosed, and the disorder may be found for the first time during pregnancy [3].

While bleeding, pruritus, and discomfort are uncommon side effects of CA, if they do occur, they are typically asymptomatic. The emergence of the lesions typically causes psychological and psychosexual anguish; therefore, patients usually present with qualms about it. Inadvertent discovery of CA may also occur during routine female gynecological exams, and the type of HPV and the site of infection determine how the lesion appears [4].

This case study features a young pregnant female with genital lesions and complications.

## Case presentation

A 20-year-old primigravida presented to the obstetrics and gynecology department in the 11th week of gestation with chief complaints of lesions over the labia majora and anus, accompanied by itching and spotting for the past six days. The symptoms had increased in severity on the morning of the presentation.

Upon general examination, the heart rate was 76 beats per minute, blood pressure was 130/90 mmHg, respiration rate was 16 breaths per minute, and the temperature was within the normal range. Auscultation revealed clean lungs in every sector. S1 and S2 were normal, with no murmurs, gallops, or rubs. Diabetes mellitus, hypertension, trauma, mouth ulcers, urinary symptoms, appetite loss, weight loss, or serious sexual sickness were not present in the past. The patient did not have a history of sexual interaction with anybody other than her spouse before or after marriage, and the husband did not have a history of the same lesions.

**Figure 1:**
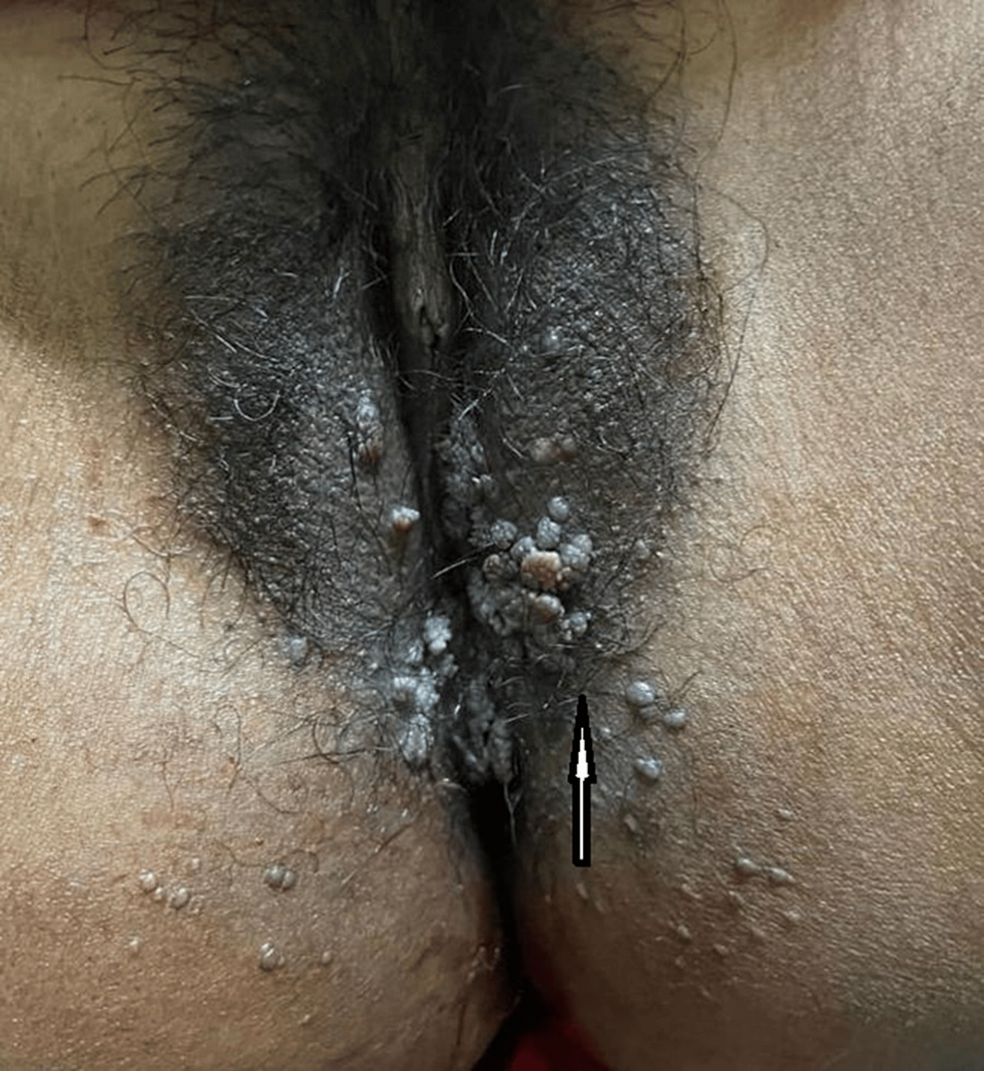
White arrow showing cauliflower-like growth, irregular and multiple in number, present over the labia majora and inner thigh.

Upon vaginal examination, bleeding through the os was observed, and lesions felt sensitive to the touch. On a peripad, tissues or blood clots were observed. The patient received an injection of antifibrinolytics, along with iron and calcium tablets. Additionally, protein powder, two tablespoons twice daily, was prescribed to control the bleeding. A dermatologist visit after the cutaneous examination revealed warts that resembled uneven growth, multiple in number, and showed signs of irritation across the labia majora. For confirmation, an HPV DNA test was conducted using a cervical sample with a DNA Papanicolaou (PAP) test cervical sampler employing the Hybrid Capture Technology method. The result was positive (ratio ≥ 1.0), leading to the diagnosis of CA (Figure 1).

There were no abnormal findings from the standard biochemical and hematological investigations. Test results for hepatitis virus subtypes, syphilis, and HIV proved negative. Antiphospholipid antibody syndrome (APLA) and toxoplasmosis, rubella, cytomegalovirus, and herpes (TORCH) tests were conducted and yielded negative results. Ultrasonography revealed a single intrauterine gestational sac with a yolk sac and fetal pole measuring the crown-rump length (CRL) of 3.5 mm; however, no cardiac activity was observed. A discrepancy of more than seven days existed between the gestational age calculated by the last menstrual period and the gestational age determined by ultrasound. The results suggested that a threatened abortion was in progress.

The patient was diagnosed with CA, or genital warts, based on clinical presentation and physical examination findings. Unfortunately, a spontaneous miscarriage happened. Subsequently, the patient was referred to the dermatology department for the management of genital warts. Given that she had already undergone an abortion, she received treatment with podophyllin. The patient was instructed to schedule routine follow-up appointments to monitor her progress and ensure the healing of genital warts. Furthermore, conversations on HPV vaccination, future pregnancy planning, and safe sexual behaviors were started.

## Discussion

In general, genital warts, also known as CA, are benign lesions that are flat, papular, or pedunculated growths on the genital mucosa. Depending on their location and size, they can also cause pain, friability, or pruritus. Pregnancy usually appears to promote the lesion's development and recurrence, which is thought to be due to the suppressed immune responses that support HPV multiplication during pregnancy as well as physiological alterations to the external genitalia. The proliferation of genital lesions during pregnancy is also attributed to increased contact between vaginal fluids and the genital skin and mucous membranes [5,6].

Genital warts are diagnosed visually, and a biopsy may be necessary to confirm the diagnosis. While trichloroacetic acid and bi-chloroacetic acid application, electrocautery, surgical excision, and laser treatment are advised during pregnancy, their effectiveness is limited, and there is a risk of miscarriage and preterm labor while undergoing treatment [7,8].

Given that the trophoblast is essential to the placentation process, there is reason to believe that papillomavirus, like other viruses, can also negatively impact the outcome of pregnancy. This suspicion is sparked by the increased incidence of infection of HPV in pregnant women and the recent proof that HPV can infect and replicate in the trophoblast [9,10].

Various treatment approaches are effective for these warts, considering factors such as the size of the lesion, the number of warts, their morphology, patient preference, and potential adverse effects. Also, the provider experience is one of the variables that may affect treatment choice. The existence of immunosuppression and therapy compliance are two factors that may influence how well a patient responds to treatment. Most individuals need multiple treatments instead of just one during their course of therapy. Due to the immune system's natural suppression during pregnancy, HPV-induced lesions typically worsen throughout pregnancy. Additionally, there's a chance of infecting the fetus during labor. Thus, in these situations, a cesarian section is the recommended procedure [11].

## Conclusions

Although the precise prevalence of CA is unknown, the illness has been recognized since prehistoric times. Both pregnant and nonpregnant women are experiencing an increase in the incidence of urogenital wart-like growths. A minor genital wart lesion may get larger and become more challenging to treat during pregnancy; however, it will gradually retract again after delivery. Due to its extensiveness and unusual appearance, there may be a diagnostic conundrum during pregnancy. Treatment should only be administered if the patient complains of symptoms such as itching or discomfort in the vaginal area, along with any other relevant complaints. This cautious approach is due to the potential for spontaneous regression of the lesion. Also, the treatment does not reduce the transmission rates. This case presented a challenging scenario where the abortion occurred due to the infection of HPV in early pregnancy, confirmed by a positive HPV DNA test. Other potential causes of abortion, including TORCH infection and APLA, were ruled out. The evidence points toward CA as the likely cause of the abortion, a rare condition in itself. Subsequently, the patient was further managed by a dermatologist.

## References

[1] (2023). Ectopic Pregnancy and Miscarriage: Diagnosis and Initial Management. https://www.nice.org.uk/guidance/cg154.

[2] Mouri Mi, Hall H, Rupp TJ (2023). Threatened Abortion. https://www.ncbi.nlm.nih.gov/books/NBK430747/.

[3] Pennycook KB, McCready TA (2023). Condyloma Acuminata. https://www.ncbi.nlm.nih.gov/books/NBK547667/.

[4] Costa-Silva M, Fernandes I, Rodrigues AG, Lisboa C (2017). Anogenital warts in pediatric population. An Bras Dermatol.

[5] Wiley DJ, Douglas J, Beutner K, Cox T, Fife K, Moscicki AB, Fukumoto L (2002). External genital warts: diagnosis, treatment, and prevention. Clin Infect Dis.

[6] Cates W Jr, American Social Health Association Panel (1999). Estimates of the incidence and prevalence of sexually transmitted diseases in the United States. Sex Transm Dis.

[7] Dinh TH, Sternberg M, Dunne EF, Markowitz LE (2008). Genital warts among 18- to 59-year-olds in the United States, national health and nutrition examination survey, 1999--2004. Sex Transm Dis.

[8] Workowski KA (2015). Centers for Disease Control and Prevention sexually transmitted diseases treatment guidelines. Clin Infect Dis.

[9] Reeves WC, Ruparelia SS, Swanson KI, Derkay CS, Marcus A, Unger ER (2003). National registry for juvenile-onset recurrent respiratory papillomatosis. Arch Otolaryngol Head Neck Surg.

[10] Baggish MS (1980). Carbon dioxide laser treatment for condylomataacuminata venereal infections. Obstet Gynecol.

[11] Devi LT, Pathania K (2009). Pregnancy with HPV associated viral warts. Med J Armed Forces India.

